# An ecological quantification of the relationships between water, sanitation and infant, child, and maternal mortality

**DOI:** 10.1186/1476-069X-11-4

**Published:** 2012-01-27

**Authors:** June J Cheng, Corinne J Schuster-Wallace, Susan Watt, Bruce K Newbold, Andrew Mente

**Affiliations:** 1 Public Health and Preventive Medicine Residency Program, Department of Clinical Epidemiology and Biostatistics, McMaster University, Hamilton, ON, Canada; 2 United Nations University Institute for Water, Environment and Health (UNU-INWEH), Hamilton, ON, Canada; 3 School of Geography and Earth Sciences, McMaster University, Hamilton, ON, Canada; 4 McMaster Institute of Environment and Health, McMaster University, Hamilton, ON, Canada; 5 School of Social Work, McMaster University, Hamilton, ON, Canada; 6 Department of Clinical Epidemiology and Biostatistics, McMaster University, Hamilton, ON, Canada; 7 Population Health Research Institute, David Braley Cardiac, Vascular, and Stroke Research Institute, Hamilton General Hospital, Hamilton, ON, Canada

**Keywords:** Water, Sanitation, Maternal health, Infant health, Child health, Millennium development goals

## Abstract

**Background:**

Water and sanitation access are known to be related to newborn, child, and maternal health. Our study attempts to quantify these relationships globally using country-level data: How much does improving access to water and sanitation influence infant, child, and maternal mortality?

**Methods:**

Data for 193 countries were abstracted from global databases (World Bank, WHO, and UNICEF). Linear regression was used for the outcomes of under-five mortality rate and infant mortality rate (IMR). These results are presented as events per 1000 live births. Ordinal logistic regression was used to compute odds ratios for the outcome of maternal mortality ratio (MMR).

**Results:**

Under-five mortality rate decreased by 1.17 (95%CI 1.08-1.26) deaths per 1000, *p *< 0.001, for every quartile increase in population water access after adjustments for confounders. There was a similar relationship between quartile increase of sanitation access and under-five mortality rate, with a decrease of 1.66 (95%CI 1.11-1.32) deaths per 1000, *p *< 0.001. Improved water access was also related to IMR, with the IMR decreasing by 1.14 (95%CI 1.05-1.23) deaths per 1000, *p *< 0.001, with increasing quartile of access to improved water source. The significance of this relationship was retained with quartile improvement in sanitation access, where the decrease in IMR was 1.66 (95%CI 1.11-1.32) deaths per 1000, *p *< 0.001. The estimated odds ratio that increased quartile of water access was significantly associated with increased quartile of MMR was 0.58 (95%CI 0.39-0.86), *p *= 0.008. The corresponding odds ratio for sanitation was 0.52 (95%CI 0.32-0.85), *p *= 0.009, both suggesting that better water and sanitation were associated with decreased MMR.

**Conclusions:**

Our analyses suggest that access to water and sanitation independently contribute to child and maternal mortality outcomes. If the world is to seriously address the Millennium Development Goals of reducing child and maternal mortality, then improved water and sanitation accesses are key strategies.

## Background

A World Health Organization (WHO) report found that almost one tenth of the global disease burden could be prevented by improving water supply, sanitation, hygiene and management of water resources [1]. Another estimate reports that 4.0% of all deaths and 5.7% of total disability-adjusted life years can be attributed to water, sanitation, and hygiene [2]. In addition, water quality and safety related to environmental chemicals adds to the considerable disease burden [3,4]. Although these reports and others have calculated disease burden related to poor water supply, sanitation, and hygiene there has not been a quantification of the improvement in health related outcomes due to improvements in water supply and sanitation. Quantification would further support the importance of investing in water and sanitation as a development strategy and would provide a mechanism for monitoring progress.

Worldwide, 1.4 million children die each year from preventable diarrheal diseases and some 88% of diarrhea cases are related to unsafe water, inadequate sanitation, or insufficient hygiene [2,5]. In addition, there are 860 000 preventable child deaths per year due to malnutrition. Childhood underweight, defined as weight two standard deviations below the mean, is implicated in 35% of all deaths of children under the age of five worldwide, or about 70 000 deaths per year [5,6]. An estimated 50% of this underweight or malnutrition is associated with repeated diarrhea or with intestinal nematode infections as a result of unsafe water, inadequate sanitation or insufficient hygiene [5]. Children also bear 54% of the burden of illness from environmental exposure to chemicals--estimated to be 4.9 million deaths in total worldwide--some of which is caused by exposure through contaminated water [3].

Pregnant women face a similar, equally dire situation, particularly because of their vulnerability to anemia, vitamin deficiency, trachoma and hepatitis, all of which can lead to increased morbidity and mortality [7]. The provision of safe water for medical purposes to treat such illness can improve newborn and child health in addition to maternal health [8]. Currently, health centres providing maternal and delivery care can expose women to unsafe water, poor sanitation and poor management of medical waste: 15% of all maternal deaths are caused by infections in the 6 weeks after childbirth and have mainly been found to be due to unhygienic practices and poor infection control during labour and delivery [9].

Despite the importance of water and sanitation and the availability of interventions, 2.6 billion people in the world currently lack access to basic sanitation, while 884 million people lack access to safe drinking water [10,11]. In order to promote access to improved water and sanitation, the Millennium Development Goals (MDG), including access to safe drinking water and basic sanitation (target 7 C), infant and child health (MDG 4), and maternal health (MDG 5) guide development and planning policies [12].The MDGs, along with suggested indicators for measure progress, are as follows:

MDG 4: Reduce child mortality

Target 4A: reduce by two thirds, between 1990 and 2015, the under-five mortality rate

Indicator 4·1: under-five mortality rate

Indicator 4·2: IMR

MDG 5: Improve maternal health

Target 5A: reduce by three-quarters, between 1990 and 2015, the MMR

Indicator 5·1: MMR

MDG 7: Ensure environmental sustainability

Target 7C: halve, by 2015, the proportion of people without sustainable access to safe drinking water and basic sanitation

Indicator 7·8: proportion of population using an improved drinking water source

Indicator 7·9: proportion of population using an improved sanitation facility [12].

Despite progress, the WHO/UNICEF Joint Monitoring Programme reported that at the current rate, the world will miss the MDG *sanitation *target by 13%, with 2.7 billion people lacking basic sanitation [11]. Even if the target is met, there will still be 1.7 billion people without access to improved sanitation [11]. More positively, the world will meet the MDG *water *target at the current rate [11]. However, even if the goal is met by 2015, there will still be 672 million people without access to improved water sources [11].

Although links have been made between water and sanitation and newborn, child, and maternal health, there have not been studies quantifying these relationships globally using country-level data [13]. Will, for example, the failure to provide water and sanitation jeopardize the achievement of MDG four and five? In this paper, we take the first steps towards quantifying the relationship between water and sanitation and infant, child, and maternal mortality: How much does safe water and sanitation contribute to these mortality outcomes on a global scale? This quantification would further enforce the importance of investing in water and sanitation as a development strategy.

## Methods

### Data source

Country level data were collected for 193 countries from several organizations including the World Health Organization Statistical Information System [14], United Nations Children's Fund Childinfo [15], and World Bank Open Data [16].

### Outcome variables

Four key outcome variables were explored in this analysis: under-five mortality rate, IMR, MMR, and proportion of under-five deaths due to diarrhea. Under-five mortality rate is defined as the probability of a child born in a specific year or period dying before reaching the age of five per 1000 live births, if subject to age-specific mortality rates of that period [17]. The proportion of under-five deaths due to diarrhea is estimated by the WHO based on the causes of death that are entered on the medical certificate of cause of death in countries and recorded by the civil (vital) registration systems. For the analyses, the concept of the 'underlying cause of death' is defined by ICD. In countries with incomplete or no civil registration, causes of death are those reported as such in epidemiological studies that use verbal autopsy algorithms to establish cause of death [18]. IMR is defined as the probability of dying between birth and exactly 1 year of age, expressed per 1 000 live births [19]. MMR is defined as number of maternal deaths per 100 000 live births during a specified time period, usually 1 year [20]. All outcome variables were obtained from the World Health Survey 2010, and the time period used for our analyses were from the year 2008.

### Independent variables

The proportion of population with access to improved water source and the proportion of population with access to improved sanitation, as defined by the WHO/UNICEF Joint Monitoring Programme, are our key predictors of interest. Data were obtained for the year 2008. The following are detailed definitions of improved access to water and sanitation:

Access to safe drinking water is measured by the percentage of the population using improved drinking-water sources.

• Improved drinking water source is a source that, by nature of its construction, adequately protects the water from outside contamination, in particular from fecal matter. Common examples:

○ Piped household water connection

○ Public standpipe

○ Borehole

○ Protected dug well

○ Protected spring

○ Rainwater collection

Access to sanitation is measured by the percentage of the population using improved sanitation facilities.

• Improved sanitation includes sanitation facilities that hygienically separate human excreta from human contact.

• Access to basic sanitation is measured against the proxy indicator: the proportion of people using improved sanitation facilities (such as those with sewer connections, septic system connections, pour-flush latrines, ventilated improved pit latrines and pit latrines with a slab or covered pit)

• Shared sanitation facilities are otherwise-acceptable improved sanitation facilities that are shared between two or more households. Shared facilities include public toilets and are not considered improved [11].

Other covariates included in the analyses are: Gross National Income (GNI) 2008 [16]; total fertility rate per woman 2008 [14]; WHO world region, adult literacy rate 2000-2008 [14]; percentage of births attended by skilled health personnel 2000-2008 [14]; percentage of women who used antenatal care provided by skilled health personnel for reasons related to pregnancy at least once during pregnancy, as a percentage of live births in a given time period 2000-2009 [14]; and deaths (1 000's) attributable to diarrheal diseases 2002 [14].

### Missing data

Due to the limited available sample size of 193 countries, attempts were made to preserve the power of the study. Percentage of missing data ranged from 0 to 32% (62/193). Missing data were imputed using a Multiple Imputation by Chained Equation (MICE) approach to obtain a full dataset [21]. See Table 1 for summary statistics of all raw variables in the study, including the number of missing values.

**Table 1:** Summary table of all variables used in regression analyses

Variable	Mean	Standard Deviation	25th percentile	50th percentile	75th percentile	Minimum value	Maximum value	Number of missing values
% access to improved water source	85.99	17.00	80	94	100	30	100	24

% access to improved sanitation	71.14	30.18	48	84	98	9	100	23

Under-five mortality rate (per 1 000 live births)	48.30	55.01	10	23	69	2	257	0

% under-five mortality due to diarrheal disease	7.15	7.26	1	5	13	0	29	0

IMR (per 1 000 live births)	33.80	33.38	9	21	53	1	165	0

MMR (per 100 000 live births)	206.60	307.66	8	44	317	0	1600	24

GNI	10790.76	16203.34	1045	3730	11940	140	87070	9

Fertility per woman	2.88	1.44	1.80	2.40	3.85	1.20	7.10	1

Adult literacy rate	80.77	19.80	71	88.50	97	26	100	57

Deaths due to diarrhea (1 000's)	8792.47	33797.29	0	300	5000	0	402200	7

% birth attended by skilled health personnel	79.80	25.62	61	94.5	100	6	100	15

% using antenatal care (at least 1 visit)	85.37	16.56	80	91	97	16	100	62

Regions of the world is a categorical variable. Its values are defined as the following: 1-African Region; 2-Europe; 3-Eastern Mediterranean; 4-Americas; 5-Southeast Asia; 6-Western Pacific

### Statistical analyses

Linear regression and ordinal logistic regression models were used. The generic model for the linear regression is as follows:

$${\text{y}}_{\text{i}}=\beta_{\text{1}}{\text{x}}_{\text{i1}}+\beta_{\text{2}}{\text{x}}_{\text{i2}}+...+\beta_{\text{p}}{\text{x}}_{\text{ip}}+\varepsilon_{\text{i}},\;\;\text{i}=\text{1},...,\text{n},$$

where y is the outcome variable, β's are the parameter coefficients, and x's are the predictor variable or covariates, and ε an error term.

The generic model for the ordinal logistic regression is as follows:

$$\text{Logit}\left[\text{P}\left(\text{y}\leq \text{j}\right)\right]=\alpha_{\text{j}}+\beta_{\text{1}}{\text{x}}_{\text{1}}+\beta_{\text{2}}{\text{x}}_{\text{2}}+\ldots +\beta_{\text{p}}{\text{x}}_{\text{p}}+\varepsilon_{\text{i}},\;\;\text{j}=\text{1},...\text{J}-\text{1},$$

where β's describe the effect of X on the log odds of response (y) in category j or below. This model assumes an identical effect of x for all j-1. In other words, it assumes proportional odds. This model was chosen for its potential for greater power and easier interpretations compared to multi-category logit models [22].

In order to satisfy the underlying assumption of linear regression, positively skewed variables including GNI, total fertility per woman, under-five mortality rate, and IMR were log-transformed to follow a symmetrical distribution. Where log transformation was not possible, quartiles were created: MMR, proportion with access to improved water source, proportion with access to improved sanitation, adult literacy rate, and percent births attended by skilled health personnel.

For continuous outcomes--log-transformed under-five mortality rate and log-transformed IMR--linear regression model was used with estimates subsequently retransformed to their natural units. For categorical outcomes--MMR quartiles and percent under-five mortality rate due to diarrhea quartiles--the ordinal logistical regression model was used. To test the assumption of proportional odds, the Stata Brant test for parallel regression was performed [23]. Predictor variables and confounders included in each analysis were based on past literature and expert opinion. Highly correlated covariates as defined by *r *> 0.8 were not included in the same equation. Akaike Information Criteria was used as an additional aid to help determine the optimal regression equation. All statistical analyses were performed using Stata 10.0.

In all cases, adjusted and unadjusted models were estimated. Unadjusted models included only the dependent variable and water (sanitation) on the right hand side. Adjusted models accounted for developmental effects. Water and sanitation were considered in separate models in this study. Due to considerable overlap between the two variables, water and sanitation access often cancelled each other in effect in test regressions. Their simultaneous effect, therefore, was not explored.

A note about variable interpretations: from this point on GNI and fertility rate per woman should be understood to be log transformed values. In addition, the following variables are presented as quartiles: percent under-five mortality due to diarrheal diseases, MMR, proportion of population with access to improved water source, proportion of population with access to improved sanitation, adult literacy rate, deaths (in thousands) due to diarrhea, and percent births attended by skilled health personnel. In ordinal logistic regression, odds ratios (OR's) represent the odds of being in a higher quartile of the outcome variable compared to a lower quartile.

## Results

In our analyses, increased access to improved water sources was significantly associated with decreased under-five mortality rate, decreased odds of under-five mortality due to diarrhea, decreased IMR, and decreased odds of MMR in our analyses (see Table 2 for univariable models and Table 3 for multivariable models and results). Under-five mortality rate was seen to decrease by 2.25 (95%CI 2.05-2.50) deaths per 1000 with increased water access in the unadjusted regression. After adjustments for GNI, fertility per woman, MMR, and region of the world as potential confounders, this decrease continued to be significant at *p *< 0.001, 1.17 (95%CI 1.08-1.26) deaths per 1000. The estimated odds ratio that increased access to water was significantly associated with increased odds of under-five child mortality due to diarrhea was 0.20 (95%CI 0.14-0.28). This odds ratio changed to 0.46 (95%CI 0.30-0.70), *p *< 0.001 in the adjusted model. IMR, in the unadjusted model, decreased by 2.12 (95%CI 1.93-2.29) deaths per 1000 with increased access to improved water source. This relationship retained its significance in the multivariable analysis, and the decrease in IMR was 1.14 (95%CI 1.05-1.23) deaths per 1000, *p *= 0.001. The estimated odds ratio that increased water access was significantly associated with increased MMR is 0.20 (95%CI 0.14-0.28). This odds ratio was 0.58 (95%CI 0.39-0.86), *p *= 0.008 in the adjusted analysis.

**Table 2:** Unadjusted Regressions

	Coefficient (95% Confidence interval (CI))	*p*-value
Predictor: proportion of population with access to improved *water source*^a^		

Under-five mortality rate (per 1 000 live births)	-2.25 (-2.50,-2.05)^b^	< 0·001

% under-five mortality due to diarrhea^a^	OR:0·20 (0·14, 0·28)^c^	< 0·001

IMR (per 1 000 live births)	-2.12 (-2.29,-1.93)	< 0·001

MMR^b ^(per 100 000 live births)	OR:0·20 (0·14, 0·28)	< 0·001

Predictor: proportion of population with access to improved *sanitation*^a^		

Under-five mortality rate (per 1 000 live births)	-2.45 (-2.66,-2.27)	< 0.001

% under-five mortality due to diarrhea^a^	OR: 0.20 (0.14, 0.28)	< 0.001

IMR (per 1 000 live births)	-2.29 (-2.48,-2.12)	< 0.001

MMR^b ^(per 100 000 live births)	OR: 0.14 (0.10, 0.20)	< 0.001

^a^variable divided into quartiles

^b^Sample interpretation: under-five mortality rate is seen to decrease by 2.25 (95%CI 2.05, 2.50) with increasing quartile of proportion of population with improved water access

^c^Sample interpretation: the estimated odds ratio that increased population quartile access to water is significantly associated with increased odds of under-five child mortality due to diarrhea is 0.20 (95%CI 0.14, 0.28)

**Table 3:** Adjusted Multivariable Regressions

	Coefficient (95% Confidence interval (CI))	*p*-value
Predictor: proportion of population with access to improved *water source*^a^		

Under-five mortality rate^b ^(per 1 000 live births)	-1.17 (-1.26,-1.08)^f^	< 0.001

% under-five mortality due to diarrhea^a, c^	OR: 0.46 (0.30, 0.70)^g^	< 0.001

IMR^b ^(per 1 000 live births)	-1.14 (-1.23,-1.05)	0.001

MMR^a, e ^(per 100 000 live births)	OR: 0.58 (0.39, 0.86)	0.008

Predictor: proportion of population with access to improved *sanitation*^a^		

Under-five mortality rate^b ^(per 1 000 live births)	-1.66 (-1.32,-1.11)	< 0.001

% under-five mortality due to diarrhea^a, c^	OR: 0.66 (0.41, 1.05)	0.08

IMR^d ^(per 1 000 live births)	-1.66 (-1.32,-1.11)	< 0.001

MMR^a, e ^(per 100 000 live births)	OR: 0.52 (0.32, 0.85)	0.009

^a^variable divided into quartiles

^b^linear regression, controlled for GNI, fertility per woman, maternal mortality ratio, region of the world

^c^ordinal logistic regression, controlled for GNI, deaths due to diarrhea (1 000 s), MMR, region of the world

^d^linear regression GNI, fertility per woman, MMR, region of the world

^e^ordinal logistic regression controlled for GNI, fertility per woman,% births attended by skilled health personnel, region of the world

^f^Sample interpretation: under-five mortality rate is seen to decrease by 1.17 (95%CI 1.08, 1.26) with increasing quartile of percent of population with improved water access

^g^Sample interpretation: the estimated odds ratio that increased population quartile access to water is significantly associated with increased odds of under-five child mortality due to diarrhea is 0.46 (95%CI 0.30, 0.70)

Increasing access to improved sanitation was significantly associated with decreased under-five mortality rate, decreased odds of under-five mortality due to diarrhea, decreased IMR, and decreased odds of MMR in our analysis. Under-five mortality rate was seen to decrease by 2.45 (95%CI 2.27-2.66) deaths per 1000 with increasing access to sanitation quartile in the unadjusted regression. After adjustments for GNI, fertility per woman, MMR, and region of the world as potential confounders, this decrease continued to be significant at *p *< 0.001, 1.66 (95%CI 1.11-1.32) deaths per 1000. See Table 2 for univariable results and Table 3 for the multivariable models and results. The estimated odds ratio that increased access to sanitation was significantly associated with increased odds of under-five child mortality due to diarrhea was 0.20 (95%CI 0.14-0.28). This odds ratio changes to 0.66 (95%CI 0.41-1.05), *p *= 0.08 in the adjusted model. IMR, in the unadjusted model, decreased by 2.29 (95%CI 2.12-2.48) deaths per 1000 with increasing access to improved sanitation. This relationship retained its significance in the multivariable analysis, and the decrease in IMR was 1.66 (95%CI 1.11-1.32) deaths per 1000, *p *< 0.001. The estimated odds ratio that increased sanitation access was significantly associated with increased MMR is 0.14 (95%CI 0.10-0.20). This odds ratio was 0.52 (95%CI 0.32-0.85), *p *= 0.009 in the adjusted analysis.

## Discussion

Our analyses show interesting and statistically significant relationships between water and maternal, infant, and child mortality. Increasing access to water and sanitation are significantly associated with decreases in the negative health outcomes of interest, namely under-five child mortality, under-five child mortality due to diarrhea, IMR, and MMR. These associations remain significant after adjusting for known and expected confounders, except in the case of under-five mortality due to diarrhea and poor access to sanitation. For the linear outcomes, under-five mortality rate and IMR, the R^2 ^value stands at 91% and 89%, respectively, for both access to water and sanitation. This allows confidence that the models account for a large part of the variability in the outcome measures. This is not to say that access to improved water and sanitation account for such high percentages of the outcome, but that in they do so in combination with all other variables. Indeed, we see that the partial R^2 ^of water access is 8.86% for under-five mortality rate, and 5.82% for IMR. Sanitation access shows partial R^2 ^of 9.23% for under-five mortality rate, and 8.38% for IMR. A similar measure cannot be calculated for ordinal logistic regression, although the regressions were highly significant as measured by the log-likelihood.

Under-five mortality rate is significantly related to increasing water access, decreasing by 1.17 deaths per 1000, after adjustments for GNI, fertility per woman, MMR, and region as potential confounders. A similar relationship is seen for sanitation access and under-five mortality rate, with a decrease of 1.66 deaths per 1000. We then performed an additional analysis of under-five mortality rate due to diarrhea since diarrhea is the second leading cause of death in children under five [5]. We found that increased access to water is significantly associated with decreased odds of under-five child mortality due to diarrhea, supporting the argument that water access is significantly associated with decreased odds of diarrhea mortality. Children are more susceptible to poor outcomes from diarrhea--such as life-threatening dehydration--in part because water makes up a larger percentage of their body weight [5]. Diarrhea can also contribute to under-nutrition through mal-absorption. Diarrhea, along with nematode infections caused by poor sanitation, can lead to 3.5 million under-five child deaths per year [5]. A World Bank publication found that in the absence of diarrhea, the nutritional status of those who are undernourished improves quickly [24]. These known relationships between water, sanitation and child health support the relationships identified in our models.

Access to water and sanitation have also been found to be related to IMR, with the IMR decreasing by 1.14 deaths per 1000 with increasing access to improved water source. This relationship retains its significance in the sanitation analysis, where the decrease in IMR was 1.66 deaths per 1000. This pattern of findings can be explained in several different ways. Access to clean water is essential for mothers who do not breastfeed; the use of dirty containers and unsafe water for formula preparation puts infants' health at risk. Infants who are not breastfed are six times more likely to die from infectious diseases, including diarrhea, in the first 2 months of life than those who are breastfed [25]. In addition, many basic birth practices, including hand washing, can affect infant mortality outcomes [26]. Unhygienic practices and poor infection control have also been shown to influence maternal mortality, which in turn will have an effect on infant mortality, as evidence shows that children who lose their mother are 10 times more likely to die before their second birthday [8,27].

The estimated odds ratio that increased water access is significantly associated with increased MMR is 0.58 in the adjusted analysis. The corresponding odds ratio for sanitation is 0.52. Both odds ratios suggest that better access to water and sanitation are associated with decreased maternal mortality. Several reasons articulated in the scientific literature may contribute to this relationship. Better water quality and sanitation help women to reduce physical burdens of carrying water, which translates into improvement in the expectant mothers' health. This improvement in maternal health in turn enables parents to provide better care to their children as they themselves are less vulnerable to diseases. In addition to being vulnerable to water and sanitation related disease such as anemia, vitamin deficiency, trachoma and hepatitis, the effects of such illnesses can be more serious. For example, anemia and associated nutritional deficiency can contribute to up to 20% of maternal deaths [28]. Maternal mortality can also be increased by exposure to unsafe water and sanitation and poor management of medical waste in health centers. Half of all infection-related deaths can be averted by hygienic childbirth techniques employed by skilled birth attendants. Both clean water and skilled birth attendants are necessary for lower maternal mortality [29].

Our study is an observational and cross-sectional study based on major databases from international sources. We have found strong associations between access to water and sanitation and child, infant, and maternal mortality on a global scale. This is supported by previous literature on smaller scales and reinforces the importance of water and sanitation access to child, infant, and maternal mortality. Due to the ecological, observational nature of the study, this relationship cannot be proven to be causal, though there is a strong suggestion of the interconnections. Such connections should be explored with longitudinal analyses to determine the causative relationships.

Ecological studies such as this one have been viewed unfavorably within the epidemiological literature. They have been criticized because of ecological fallacy and the problem of geographic scale, the lack of statistical rigor, and the inability to appropriately define risks or health measures [30,31]. However, despite the disadvantages of ecologic studies, there are also a number of advantages, including the ability to study a large population and to address questions of environmental health before turning to expensive and intensive case-control or cohort studies [30]. For example, ecological studies of mortality and morbidity at the regional scale (including counties) have demonstrated important findings, while generating new avenues of research at the same time [30,32-38]. In particular, ecologic studies provide a relatively inexpensive way to examine regional variations in the health of large populations. While the authors fully recognize the weaknesses of this approach, there is significant value to this type of analysis in determining the relationships studied on a global scale. The associations between water and sanitation and health outcomes need to be further elucidated with longitudinal approaches and controlled studies. Other types of analyses were considered, such as a time series comparison. However, with the large amount of missing data on the global scale, the level of uncertainty added by other approaches was considered by our group to be too large. With current available data, this approach was considered the most suitable and the best available by the authors for the research question posed.

An issue we were not able to address in our models is the rural-urban divide. The known differences in water and sanitation access, as well as mortality outcomes between rural and urban areas cannot be examined through country-level analysis. These differences may lead to bias in our calculations, especially in countries where rural and urban populations experience large differences in the indicators we used. Similar issues exist for other types of heterogeneity within a country--such as gender, race, and cultural differences. Lastly, we did not assess water quality and safety related to environmental chemicals (eg, exposure to arsenic from drinking water in deep wells). While exposure to toxic chemicals in drinking water adds further to the considerable disease burden [3,4], this area was beyond the scope of our study. One element that is crucial to this type of analysis is the quality of the data collected by the various countries. Does access to improved water source mean safe water? What does safe water really mean? Are the current measurements of water access the most useful? The rapid assessment of drinking-water quality (RADWQ) measurement is an alternative to the current measurement of access. However, RADWQ is not economically viable to perform globally, and carries with it opportunity costs [39]. A cost-effective and meaningful measurement for safe water is yet to be determined. Sanitation indicators are even more difficult to measure. The current standard for improved sanitation measures access, not use. Both water and sanitation indicators are only estimates of what we hope to measure. Similarly, there are numerous impediments to having an accurate assessment of MMR, as noted in a recent article by Hogan et al. [40], while infant and child mortality data are often lacking or have very low coverage for countries with high mortality rates [41,42]. New tools and methods need to continue to be developed for better measure and monitor the global situation of water, sanitation, and infant, child and maternal mortality outcomes; our analysis along with past literature strongly support the existence of such relationships.

## Conclusions

Our analyses show interesting and statistically significant relationships between access to water and sanitation and maternal, infant, and child mortality. With this knowledge, plans for development should include improving water and sanitation access. This will allow fuller achievement in MDGs four and five--improving child and maternal health.

## Abbreviations

MMR: Maternal mortality ratio; IMR: Infant mortality rate; WHO: World Health Organization; UN: United Nations; UNICEF: United Nations Children's Fund; DG: Millennium Development Goal; GNI: Gross national income; OR: Odds ratio; RADWQ: Rapid assessment of drinking-water quality.

## Competing interests

The authors declare that they have no competing interests.

## Authors' contributions

JJC was the primary writer, data collector, analyst, and interpreter. CSW assisted with data collection, interpretation and writing. SW assisted with data interpretation and writing. KBN assisted with data interpretation and writing. AM assisted with data analysis. All authors read and approved the final manuscript.

## References

[B1] Pruss-Ustun A Bos R Gore F Bartram J Safer Water, Better Health: Costs, Benefits and Sustainability of Interventions to Protect and Promote Health 2008 Geneva: WHO

[B2] Pruss A Kay D Fewtrell L Bartram J Estimating the burden of disease from water, sanitation, and hygiene at a global level Environ Health Perspect 2002 110 537 542 10.1289/ehp.02110537 12003760 PMC1240845

[B3] Pruss-Ustun A Vickers C Haefliger P Bertollini R Knowns and unknowns on burden of disease due to chemicals: a systematic review Environ Health 2011 10 9 10.1186/1476-069X-10-9 21255392 PMC3037292

[B4] Chowdhury UK Biswas BK Chowdhury TR Samanta G Mandal BK Basu GC Chanda CR Lodh D Saha KC Mukherjee SK Roy S Kabir S Quamruzzaman Q Chakraborti D Groundwater arsenic contamination in Bangladesh and West Bengal, India Environ Health Perspect 2000 108 393 397 10.1289/ehp.00108393 10811564 PMC1638054

[B5] UNICEF and WHO Diarrhea: Why Children are Still Dying and What Can be Done 2009 New York and Geneva: UNICEF and WHO

[B6] De Onis M Blossner M Borghi E Frongillo EA Morris R Estimates of global prevalence of childhood underweight in 1990 and 2015 JAMA 2004 291 2600 2606 10.1001/jama.291.21.2600 15173151

[B7] WHO/UNICEF Joint Monitoring Programme for Water Supply and Sanitation Water for Life: Making it Happen 2005 New York and Geneva: WHO and UNICEF

[B8] United Nations World Water Assessment Programme Water for the Millennium Development Goals--Why Managing Water Resources Wisely is Key to Achieving the MDGs 2010 Paris: United Nations

[B9] Goodburn E Campbell O Reducing maternal mortality in the developing world: sector-wide approaches may be the key BMJ 2001 322 917 920 10.1136/bmj.322.7291.917 11302911 PMC1120077

[B10] Fewtrell L Kaufmann RB Kay D Enanoria W Haller L Colford JM Jr Water, sanitation, and hygiene interventions to reduce diarrhea in less developed countries: a systematic review and meta-analysis Lancet Infect Dis 2005 5 42 52 10.1016/S1473-3099(04)01253-8 15620560

[B11] UNICEF and WHO Joint Monitoring Programme for Water Supply and Sanitation Progress on Drinking Water and Sanitation 2010 New York and Geneva: UNICEF and WHO

[B12] United Nations Official list of MDG Indicators http://mdgs.un.org/unsd/mdg/Host.aspx?Content=Indicators/OfficialList.htm

[B13] Yardley S Joining the Dots: Why Better Water, Sanitation and Hygiene are Necessary for Progress on Maternal, Newborn and Child Health 2010 Teddington: Tearfund

[B14] WHO Statistical Information System (WHOSIS) World health statistics 2010 http://www.who.int/whosis/whostat/2010/en/index.html

[B15] UNICEF Childinfo The state of the world's children 2010 http://www.childinfo.org/statistical_tables.html

[B16] World Bank Open Data http://data.worldbank.org/

[B17] WHOSIS Probability of dying (per 1000) under age 5 years (under-5 mortality rate) http://www.who.int/whosis/indicators/mortalityunder5/en/index.html

[B18] WHOSIS Causes of death among children aged less than 5 years (percentage of total) http://www.who.int/whosis/indicators/mortcauseslessthan5years/en/index.html

[B19] UNICEF Basic indicators http://www.unicef.org/infobycountry/stats_popup1.html

[B20] WHOSIS Maternal mortality ratio (per 100 000 live births) http://www.who.int/whosis/indicators/maternalmortratio/en/index.html

[B21] Royston P Multiple imputation of missing values: Further update of ice, with an emphasis on categorical variables Stata J 2009 9 466 477

[B22] Agresti A An Introduction to Categorical Data Analysis 1996 New York: John Wiley and Sons, Inc

[B23] UCLA Academic Technology Services, Statistical Consulting Group Stata data analysis examples ordinal logistic regression http://www.ats.ucla.edu/stat/stata/dae/ologit.htm

[B24] World Bank Environmental Health and Child Survival: Epidemiology, Economics, Experiences 2008 Washington: World Bank

[B25] WHO Collaborative Study Team on the Role of Breastfeeding on the Prevention of Infant Mortality Effect of breastfeeding on infant and child mortality due to infectious diseases in less developed countries: a pooled analysis Lancet 2002 355 451 455 10841125

[B26] Rhee V Mullany LC Khatry SK Katz J LeClerq SC Darmstadt GL Tielsch TM Maternal and birth attendant hand washing and neonatal mortality in southern Nepal Arch Pediatr Adolesc Med 2008 162 603 608 10.1001/archpedi.162.7.603 18606930 PMC2587156

[B27] Unite Nations Population Fund Giving Birth Should not be a Matter of life and Death 2007 New York: United Nations

[B28] WHO Water-related diseases http://www.who.int/water_sanitation_health/diseases/anemia/en/

[B29] Water Supply and Sanitation Collaborative Council For her, it's the Big Issue: Putting Women at the Centre of Water Supply, Sanitation and Hygiene Evidence Report 2006 Geneva: Water Supply and Sanitation Collaborative Council22288057

[B30] Walter SD The ecologic method in the survey of environmental health: 1. overview of the method Environ Health Perspect 1991 94 61 65 1954942 10.1289/ehp.94-1567938 PMC1567938

[B31] Richardson S Monfort C Elliott P, Wakefield JC, Best NG, Briggs DL Ecological correlation studies Spatial Epidemiology: methods and applications 2000 London: Oxford University Press 205 220

[B32] Griffith J Riggan WB Duncan RC Pellom AC Cancer mortality in US counties with hazardous waste sites and ground water pollution Arch Environ Health 1989 44 69 74 10.1080/00039896.1989.9934378 2930248

[B33] Walter SD Assessing spatial variations in disease rates Stat Med 1993 12 1885 1894 10.1002/sim.4780121914 8272668

[B34] Walter SD Birnie SE Marrett LD Taylor SM Reynolds D Davies J Drake JJ Hayes M The geographic variation of cancer incidence in Ontario Am J Public Health 1994 84 367 376 10.2105/AJPH.84.3.367 8129051 PMC1614820

[B35] Jerrett M Eyles J Cole D Reader S Environmental equity in Canada: an empirical investigation into the income distribution of pollution in Ontario Environ Plan A 1997 29 1777 1800 10.1068/a291777

[B36] Curriero F Heiner KS Samet J Zeger S Strug L Patz JA Temperature and mortality in 11 cities of the Eastern United States Am J Epidemiol 2002 155 80 87 10.1093/aje/155.1.80 11772788

[B37] Burnett RT Goldberg MS Size-fractionated particulate mass and daily mortality in eight Canadian cities 2003 Boston: Health Effects Institute 85 89 In revised analyses of time-series studies of air pollution and health special report.

[B38] Kaiser R Le Tertre A Schwartz J Gotway CA Daley WR Rubin CH The effect of the 1995 heat wave in Chicago on all-cause and cause-specific mortality Am J Public Health 2007 97 S158 S162 10.2105/AJPH.2006.100081 17413056 PMC1854989

[B39] WHO Report of the WHO/UNICEF joint monitoring programme: progress on sanitation and drinking water 2010 update http://www.who.int/water_sanitation_health/monitoring/key_terms/en/

[B40] Hogan MC Foreman KJ Naghavi M Ahn SY Wang M Makela SM Lopez AD Lozano R Murray CJ Maternal mortality for 181 countries, 1980-2008: a systematic analysis of progress towards Millennium Development Goal 5 Lancet 2010 375 1609 1623 10.1016/S0140-6736(10)60518-1 20382417

[B41] WHO Measuring child mortality http://www.who.int/child_adolescent_health/data/children/en/index.html

[B42] Boschi-Pinto C Velebit L Shibuya K Estimating child mortality due to diarrhoea in developing countries Bull World Health Organ 2008 86 710 717 10.2471/BLT.07.050054 18797647 PMC2649491

